# Case-control study of *ADARB1* and *ADARB2* gene variants in migraine

**DOI:** 10.1186/s10194-015-0511-y

**Published:** 2015-04-03

**Authors:** Claudia F Gasparini, Heidi G Sutherland, Bridget Maher, Astrid J Rodriguez-Acevedo, Elhame Khlifi, Larisa M Haupt, Lyn R Griffiths

**Affiliations:** Menzies Health Institute Queensland, Griffith University Gold Coast, Parklands Drive, Southport, QLD 4222 Australia; Genomics Research Centre, Institute of Health and Biomedical Innovation, Queensland University of Technology, Musk Ave, Kelvin Grove, QLD 4059 Australia; UCL Institute of Neurology, Department of Clinical and Experimental Epilepsy, Queen Square, London, WC1N 3BG UK

**Keywords:** Migraine, Migraine with aura, Migraine without aura, RNA editing, pGWAS, Norfolk Island, *ADARB1*, *ADARB2*, Headache

## Abstract

**Background:**

Migraine causes crippling attacks of severe head pain along with associated nausea, vomiting, photophobia and/or phonophobia. The aim of this study was to investigate single nucleotide polymorphisms (SNPs) in the adenosine deaminase, RNA-specific, B1 (*ADARB1*) and adenosine deaminase, RNA specific, B2 (*ADARB2*) genes in an Australian case–control Caucasian population for association with migraine. Both candidate genes are highly expressed in the central nervous system and fit criteria for migraine neuropathology. SNPs in the *ADARB2* gene were previously found to be positively associated with migraine in a pedigree-based genome wide association study using the genetic isolate of Norfolk Island, Australia. The *ADARB1* gene was also chosen for investigation due to its important function in editing neurotransmitter receptor transcripts.

**Methods:**

Four SNPs in *ADARB1* and nine in *ADARB2* were selected by inspecting blocks of linkage disequilibrium in Haploview for genotyping using either TaqMan or Sequenom assays. These SNPs were genotyped in two-hundred and ninety one patients who satisfied the International Classification of Headache Disorders-II 2004 diagnostic criteria for migraine, and three-hundred and fourteen controls, and PLINK was used for association testing.

**Results:**

Chi-square analysis found no significant association between any of the SNPs tested in the *ADARB1* and *ADARB2* genes in this study and the occurrence of migraine.

**Conclusions:**

In contrast to findings that SNPs in the *ADARB2* gene were positively associated with migraine in the Norfolk Island population, we find no evidence to support the involvement of RNA editing genes in migraine susceptibility in an Australian Caucasian population.

## Background

The International Headache Society (IHS) defines migraine as a recurrent headache disorder typified by painful attacks lasting 4–72 hours [1]. Classification criteria recognize two types of migraine, migraine with aura (MA) and migraine without aura (MO) with the former accompanied by visual or auditory disturbances [1]. Migraine generally affects 12% of the Caucasian population showing up more often in females, a statistic correlated with instability of ovarian hormones [2]. Migraine has a major impact on the wellbeing and quality of life of sufferers and their families in part due to days missed from the workplace and higher direct health-care costs [3]. Numerous theories and models regarding migraine mechanisms have emerged; the most accepted opinion is that a combination of both vascular and neural events is involved in the initiation and perpetuation of a migraine attack [4,5]. Primarily the generation of pain is attributed to complex processes within the nervous system that activate the trigeminovascular system consisting of the trigeminal nerve and its ramifications which regulate cerebral blood flow and the release of inflammatory molecules. The fact that the diameter of cranial blood vessels along with other functions is controlled by signals transmitted by nerves is further evidence to support a neurogenic theory. The aura on the other hand, occurs in only a minority of migraineurs and has been attributed to an electrophysiological phenomenon, first reported in epilepsy, termed Cortical Spreading Depression (CSD) [6].

Interactions between the environment and the genotype are important in shaping the migraine phenotype. Population based twin studies have confirmed a genetic influence ranging from 0.34 to 0.57 [7]. Genetic studies of a rare, and more severe migraine subtype, Familial Hemiplegic Migraine (FHM), have identified mutations in three causal genes that code for ion channels involved in neuronal signalling and have provided hypotheses applicable to common migraine [8]. Functional studies in cellular and animal models of mutant alleles provide direct evidence for neuronal hyperexcitability as one cellular mechanism underlying headache or aura in FHM [9]. For the most part, genes causing more common types of migraine have been identified from neurological, vascular and hormonal pathways and in 2010 the first functional variant to show linkage to familial MA was identified in *TRESK*, a potassium channel involved in neuronal excitability [10]. While genome wide association studies (GWAS) have shed new light on the types of genes involved in migraine susceptibility, many candidate gene association studies have focused on neurotransmitter-related pathways, as these pathways are considered to play a significant role in the migraine process. Therefore genes affecting synthesis and activity of neurotransmitters, including RNA editing genes, are potential candidates for involvement in migraine susceptibility.

**Figure 1:**
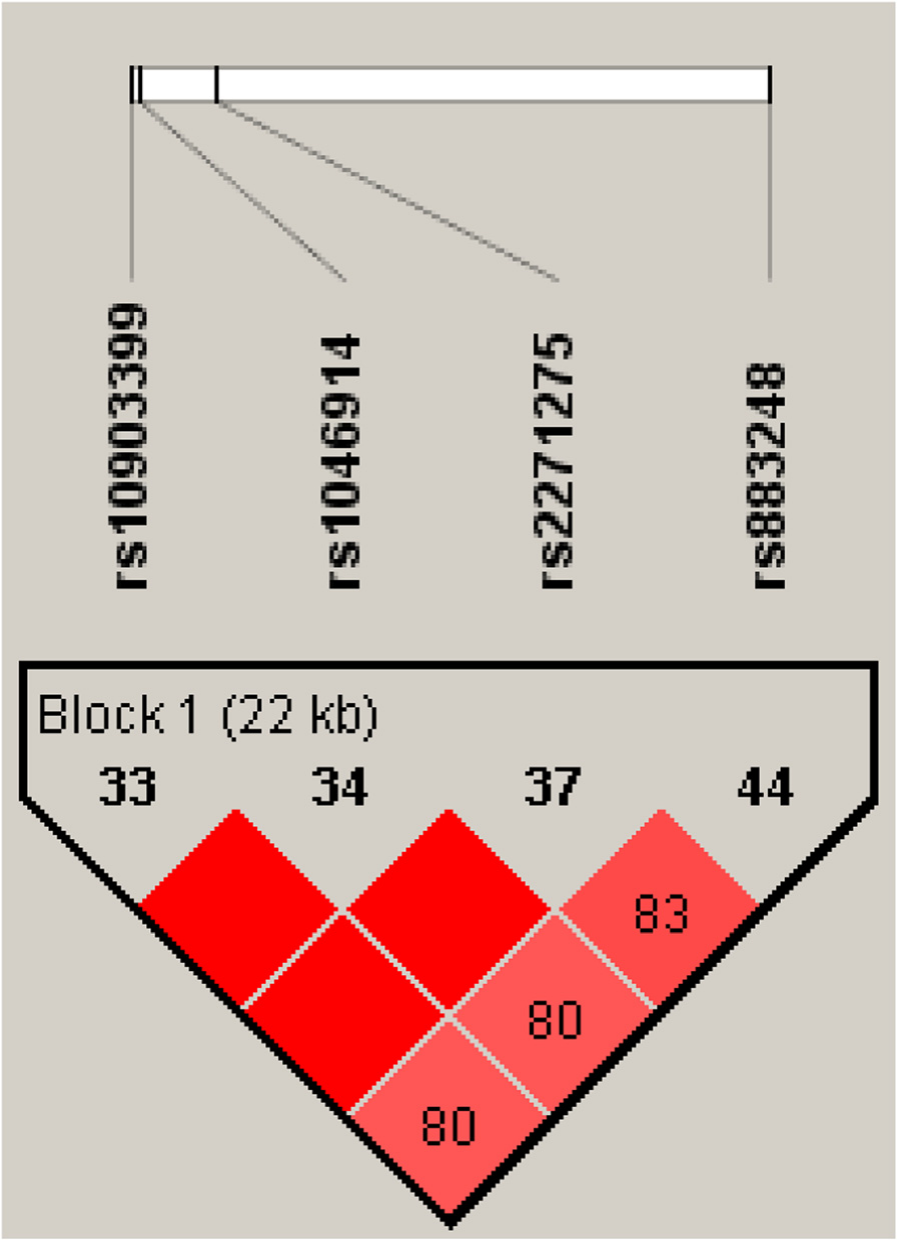
**Haplotype block of the 4 SNPs implicated in the ADARB2 gene in the Norfolk Island pGWAS sourced from Cox et al., 2012 [** 11 **].**

Isolated founder populations offer several advantages over mainstream (outbred) populations for genomic studies of disease, as both environmental noise and genetic heterogeneity are reduced. We have used the population of Norfolk Island, an island off the east coast of Australia, for genetic studies on migraine as it has well-documented family histories and an increased prevalence of the disorder [11]. We previously identified four SNPs forming a 22 kb haplotype block in *ADARB2* (Table 1 and Figure 1) that were positively associated with migraine susceptibility in a pedigree-based GWAS (pGWAS) of the population of Norfolk Island [11]. *ADARB2* is a member of the double-stranded RNA adenosine deaminase family of RNA-editing enzymes [12]. The enzymatic activity of ADARs leads to the chemical modification of Adenosine-to-Inosine (A-to-I) in specific coding regions which are then translated as guanosines by the cell’s translational machinery [13]. A-to-I RNA editing is a post-transcriptional process that permanently alters the nucleotide sequence of an RNA molecule resulting in the synthesis of proteins not encoded by the original gene sequence [14]. This is a form of chemical recoding that changes specific amino acid residues and alters the biological function of translated molecules, which is most clearly demonstrated by an alteration in channel properties including the Ca^2+^ permeability of glutamate receptors (GluRs) [15]. Perturbed A-to-I RNA editing has been implicated in human cancer, and viral infections and neurodegenerative/neurological diseases such as dyschromatosis symmetrica hereditaria (DSH), amyotrophic lateral sclerosis (ALS), Alzheimer’s disease, and Huntington’s disease epilepsy, depression and schizophrenia [16,17]. In ALS inefficient RNA editing fails to substitute an arginine for a glutamine residue in the GluR2 Q/R site of glutamate AMPA receptors in the spinal motor neurons and is proposed as a mechanism responsible for motor neuron death [18].

**Table 1:** **SNPs in** ***ADARB2*** **associated with migraine in the Norfolk Island pGWAS adapted from** [11]

**Locus**	**Gene**	**No. SNPs in Gene**	**NCBI dbSNP Ref No.**	**NCBI build 37.1 position (BP)**	**Function**	**Minor/Major allele**	**MAF**	**Beta**	**P Value**
10p15	ADARB2	4	rs10903399	1227868	Downstream	C/T	0.330	0.64	7.68E − 05
			rs1046914	1228206	3Prime UTR	G/A	0.328	0.67	3.43E − 05
			rs2271275	1230968	Non-synon	G/A	0.368	0.65	2.67E − 05
			rs883248	1250184	Intronic	G/A	0.439	0.67	3.83E − 06

*ADARB2* (also known as *ADAR3*) comprises 10 exons spanning a genomic region of 9.5 kb on chromosome 10p15.3 and encodes a catalytically inactive protein, expressed in brain, amygdala and thalamus [12]. One SNP rs2271275 confers a (Thr-Ala) amino acid change in the protein structure and has previously been associated with early-onset obsessive-compulsive disorder in some American families in a genome-wide linkage scan [19]. In addition to *ADARB2* SNPs, the Norfolk Island pGWAS also found two SNPs in the glutamate receptor, metabotropic 7 (*GRM7*) gene and one SNP in the 5-hydroxytryptamine serotonin receptor 7 adenylate cyclase-coupled (*HTR7*) gene to be associated with migraine [11]. Collectively, association of variants in these neurotransmitter-related genes *ADARB2*; *GRM7*; *HTR7* connected by a common neurological pathway supports current theories of a perturbed serotonin and glutamate mechanism in migraine and in the Norfolk pedigree. Previous studies have reported positive associations of SNPs in various neurotransmitter-related genes, e.g. *DBH* [20] and *SLC6A3* [21], with migraine in a number of case–control populations.

Considering the association of *ADARB2* with migraine in the Norfolk Island pGWAS we decided to investigate SNPs in this gene, as well as another RNA editing gene, *ADARB1*, in an Australian migraine case–control population. *ADARB1* encodes an RNA editing enzyme, expressed mainly in the central nervous system (CNS), which is involved in the downstream regulation of neurotransmitters [22]. The gene is of potential interest with respect to migraine susceptibility as the glutamate and serotonin receptor gene RNAs are the predominant substrates modified by *ADARB1* adenosine deamination [22]. *ADARB1* (also known as *ADAR2*) spans a genomic region of 25 kb on chromosome 21q22.3 and comprises 16 exons and regulates its own expression through self-editing [23]. In this study we genotyped four SNPs in *ADARB1* and nine SNPs in *ADARB2* using either TaqMan or Sequenom assays to investigate their involvement in migraine.

## Methods

### Case–control population

The study was approved by the Griffith University Ethics Committee for experimentation in humans. The study population was composed of 291 cases and 314 controls and demographical characteristics of the population are reported in (Table 2). Affected individuals were diagnosed as having MA or MO by an experienced clinical neurologist based on responses provided in a validated medical questionnaire in accordance with criteria determined by the International Headache Society (IHS) [1]. The migraine population consisted of individuals of Caucasian origin, recruited from the South East Queensland Region of Australia. The control population samples were obtained via the Genomics Research Centre Clinic, Southport and had no history of personal or familial migraine. Signed informed consent was obtained from all patients before participation in the study.

**Table 2:** **Demographical characteristics of Migraine Cases and Controls**

**Parameter**	**Cases (n = 291)**	**Controls (n = 314)**
Age: Mean (range)	46.4 (−18-67)	44.4 (18–63)
Sex		
Male	70	89
Female	221	225
MA	227	N/A
MO	64	N/A

MA - migraine with aura, MO - migraine without aura, N/A - not applicable.

### SNP selection

HapMap CEU (Utah residents with Northern and Western European ancestry) SNP genotype data was downloaded for each candidate gene and a total of 8 haplotype blocks were identified in the *ADARB1* gene and 56 haplotype blocks in the *ADARB2* gene in the program Haploview v4.2 [24]. The SNPs genotyped in the ADARB1 and ADARB2 genes were selected in different LD block, were mostly located in intronic regions of each gene and were selected to obtain reasonable coverage across the entire length of the gene (Tables 3 and 4).

**Table 3:** **TaqMan Assay and SNP information**

**TaqMan Assay ID**	**Gene**	**SNP ID**	**Position**	**SNP Type**	**Allele Change**	^**a**^ **CEU MAF**
C__15831699_10	ADARB1	rs2838771	Chr.21: 46501576	Transversion Substitution	C:G	G = 0.41
C_1211569_1_	ADARB1	rs1051367	Chr.21: 46641968	Silent Mutation	A:G	G = 0.47
C_15959830_10	ADARB2	rs2271275	Chr.10: 1230968	Missense Mutation	C:T	C = 0.34
C_32118695_20	ADARB2	rs10903467	Chr.10: 1535739	Intron, Transition Substitution	C:T	T = 0.44
C__30856132_20	ADARB2	rs11250642	Chr.10: 1622644	Intron, Transition Substitution	C:T	T = 0.50

^a^CEU MAF = HapMap Caucasian Minor Allele Frequency.

**Table 4:** **SNPs genotyped in** ***ADARB1*** **and** ***ADARB2*** **by Sequenom**

**Gene**	**SNP ID**	**Forward, Reverse and Extension Primer Sequences**	**Position**	**SNP type**	**Allele Change**	^**a**^ **CEU MAF**
**ADARB1**	rs407133	F: 5’ ACGTTGGATGCCTGCTCTGCAGTAATGAAC 3’	45396317	Intron	C:G	G 0.46
		R: 5’ ACGTTGGATGTCCTCCTCTCTTAACTCACG 3’				
		E: 5’ TCGGACCAATGCTGA 3’				
	rs422720	F: 5’ ACGTTGGATGGGAAGCAGTACATGTTCATTG 3’	45403382	Intron	A:C	C 0.36
		R: 5’ ACGTTGGATGGACTAATGCAGATGATCACC 3’				
		E: 5’ GGGTCAGTACATGTTCATTGTAAGAATT 3’				
**ADARB2**	rs3793733	F: 5’ ACGTTGGATGCAACTCCATGTCAAAAGTGC 3’	1411326	Missense	G:A	A 0.01
		R: 5’ ACGTTGGATGATGCCAGGACTCAGGTGCTT 3’				
		E: 5’CAGGAAGCATATTGTCAACCTTCCTC 3’				
	rs7070629	F: 5’ ACGTTGGATGGATGAAAAAAGGATGCCATAC 3’	1414938	Intron	G:A	A 0.37
		R: 5’ ACGTTGGATGCCTGTGATGCAGCTTCTCCT 3’				
		E: 5’ ACCTTTGATGCAGCTTCTCCTGGCACA 3’				
	rs10903479	F: 5’ ACGTTGGATGAGAACGCAATGCACTCTTCC 3’	1581881	Intron	A:T	T 0.40
		R: 5’ ACGTTGGATGTCGGTTTTGGAGTCTAGAGG 3’				
		E: 5’ CCTCATTGTCACAGAGT 3’				
	rs7094094	F: 5’ ACGTTGGATGTCTAGAAAATGCAGAAGGG 3’	1652840	Intron	T:G	G 0.38
		R: 5’ ACGTTGGATGCTTGGGCTATACTTTTTGTG 3’				
		E: 5’CATTCAGTTGTTCTAATATTATATTGA 3’				
	rs10903520	F: 5’ ACGTTGGATGTTACTCCTTAAGTGGAAGGG 3’	1672481	Intron	G:A	A 0.39
		R: 5’ ACGTTGGATGACCATGATATCTACCCCTCC 3’				
		E: 5’ AAAGAAGCAGGCGTT 3’				
	rs884861	F: 5’ ACGTTGGATGGAAAAACAGATAGACAAAGC 3’	1765662	Intron	C:G	C 0.43
		R: 5’ ACGTTGGATGCCCTGGAATAACTTCAGGGT 3’				
		E: 5’ ACAGATAGACAAAGCAGAATATAT 3’				

^a^CEU MAF = HapMap Caucasian Minor Allele Frequency.

### Genotyping methods

Genomic DNA was extracted from peripheral blood samples using a salting out method as described by Miller et al. [25]. DNA was quantified and normalised to a concentration of 20 ng/μL for genotyping experiments. Nine SNPs were genotyped as part of a Sequenom plex, which allows high throughput multiplexing of the assays into a single well. Not all SNPs are compatible with the restraints required for primer design for this method, or can be run together so therefore we also used TaqMan assays to genotype five of the SNPs. For each SNP, Sanger sequencing of a subset of samples was performed on a ABI3500 (Life Technologies, Carlsband, CA, USA) to confirm the genotypes.

### Genotyping by TaqMan

SNPs rs2838771, rs1051367 in *ADARB1* and SNPs rs2271275, rs10903467, rs11250642 in *ADARB2* were genotyped using TaqMan® SNP Genotyping Assays from Applied Biosystems (Life Technologies, Carlsband, CA, USA). Detailed information regarding SNPs genotyped in the *ADARB1* and *ADARB2* genes and a summary of assay conditions for each SNP are listed in Table 3. The genotyping protocol for each marker was exactly the same except for the use of the specific Primer-Probe Mix. The final optimized PCR reaction conditions consisted of 20 ng of genomic DNA template, TaqMan Universal PCR Master Mix (1X), SNP genotyping assay probe-primer mix (20X), DNase-free water in a 5 μL reaction volume. The PCR thermocycling conditions consisted of one cycle at 95°C for 10 min, followed by 40 cycles at 95°C for 15 s, 60°C for 1 min. The data were acquired during the annealing step and analysed using the 7900 system Sequence Detection System software (Applied Biosystems, Life Technologies Corporation) in a 384-well plate format. Nuclease-free water was used as a negative control and DNA for each genotype included as positive controls.

#### Genotyping by Sequenom

SNPs in the *ADARB1* and *ADARB2* genes were genotyped using the Sequenom MassARRAY system 4 platform, and Typer 4.0 software was used to carry out all genotyping work (MALDI-TOF; MassARRAY system, Sequenom Inc., San Diego, CA, USA). The primers for PCR and iPLEX reactions were designed using the online Assay design suite 1.0 Sequenom software (available at: www.mysequenom.com/Home) and obtained from IDT (see Table 4) (Integrated DNA Technologies, Carolville, Iowa, USA). PCR and extension reactions were performed in a 96-well plate according to the manufacturer’s instructions, using Sequenom reagents. Completed genotyping reactions were spotted in nanoliter volumes onto a matrix arrayed silicon chip with 96 elements (Sequenom SpectroCHIP) using the MassARRAY Nanodispenser. Spectro CHIPs were analyzed using the Bruker Autoflex MALDI-TOF mass spectrometer and the spectra were processed using the SpectroTYPER software (Sequenom) to yield genotypes.

### Statistical analysis

Hardy-Weinberg Equilibrium (HWE) was calculated for each SNP in case and control groups to detect deviation from the normal genotype distribution in the population. Chi-square (*χ*^2^) analysis was performed on SNPs in HWE to determine if significant differences existed in the genotype and allele frequencies in the migraine population versus controls. This analysis was completed in the software PLINK v1.07 [26]. Power estimates indicated that if the SNPs were to confer at least a two-fold increase in relative risk of migraine, the case and control groups used in this study are of sufficient size to have approximately 80% power to detect an association as statistically significant at the 0.05 level.

## Results

The aim of this study was to determine if SNPs in the *ADARB1* and *ADARB2* genes contribute to migraine susceptibility in an Australian case–control cohort. In this study, the following 4 SNPs in the *ADARB1* gene: rs2838771, rs407133, rs422720, rs1051367, and the following 8 SNPs in the *ADARB2* gene: rs3793733, rs7070629, rs10903467, rs10903479, rs11250642, rs7094094, rs10903520, rs884861 were in HWE and analysed for association. Genotypic and allelic distributions for all 12 SNPs genotyped are shown in Table 5. Minor Allele Frequencies (MAF) obtained in this study were consistent with expected allele frequencies in HapMap–Utah residents with Northern and Western European ancestry (CEU) populations. *χ*^2^ analysis was performed for migraine cases versus controls. As Table 5 shows, for the majority of SNPs genotype and allele frequencies do not differ significantly between migraineurs and controls. An allelic p-value of 0.05 was observed for rs884861. However, the result for rs884861 is not significant with Bonferroni correction for multiple testing which gives a corrected p-value threshold of 0.0042. Thus overall there was no significant association between migraine and the *ADARB1* and ADARB2 SNPs investigated.

**Table 5:** **Genotypic and allelic frequencies of migraine cases and controls and p-values derived from** ***χ*** ^**2**^ **analysis for SNPs investigated in the** ***ADARB1*** **and** ***ADARB2*** **genes**

**SNP**	**Group**	**Genotypes**			**Total (n=)**	**Genotypic p-value**	^**a**^ **MAF**	**Allelic p-value**	**Hap-Map CEU** ^**a**^ **MAF**
***ADARB1***		**CC**	**GC**	**GG**					
**rs2838771**	**Cases**	0.429	0.441	0.130	261	0.86	0.35	0.65	
	**Controls**	0.405	0.461	0.134	247		0.36		G allele, 0.36
		**GG**	**GA**	**AA**					
**rs407133**	**Cases**	0.178	0.519	0.303	264	0.95	0.44	0.35	
	**Controls**	0.179	0.529	0.292	291		0.44		G allele, 0.44
		**CC**	**CA**	**AA**					
**rs422720**	**Cases**	0.157	0.498	0.345	255	0.64	0.42	0.35	
	**Controls**	0.138	0.480	0.382	275		0.38		C allele, 0.38
		**AA**	**AG**	**GG**					
**rs1051367**	**Cases**	0.277	0.530	0.193	274	0.53	0.46	0.40	
	**Controls**	0.320	0.493	0.187	300		0.43		G allele, 0.43
***ADARB2***		**AA**	**GA**	**GG**					
**rs3793733**	**Cases**	0.0	0.017	0.983	239	^b^0.32	0.008	0.32	
	**Controls**	0.0	0.031	0.969	228		0.015		A allele, 0.009
		**AA**	**GA**	**GG**					
**rs7070629**	**Cases**	0.138	0.466	0.395	253	0.70	0.37	0.41	
	**Controls**	0.125	0.444	0.430	279		0.35		A allele, 0.37
		**TT**	**CT**	**CC**					
**rs10903467**	**Cases**	0.183	0.470	0.347	251	0.16	0.42	0.11	
	**Controls**	0.205	0.524	0.271	288		0.47		T allele, 0.48
		**TT**	**TA**	**AA**					
**rs10903479**	**Cases**	0.195	0.489	0.316	266	0.33	0.44	0.15	
	**Controls**	0.153	0.490	0.357	294		0.40		T allele, 0.40
		**TT**	**CT**	**CC**					
**rs11250642**	**Cases**	0.200	0.459	0.341	255	0.34	0.42	0.31	
	**Controls**	0.200	0.500	0.300	270		0.45		T allele, 0.45
		**GG**	**GT**	**TT**					
**rs7094094**	**Cases**	0.100	0.420	0.480	246	0.83	0.37	0.62	
	**Controls**	0.104	0.442	0.454	269		0.33		G allele, 0.37
		**AA**	**GA**	**GG**					
**rs10903520**	**Cases**	0.114	0.493	0.393	280	0.55	0.36	0.62	
	**Controls**	0.144	0.476	0.380	290		0.38		A allele, 0.38
		**GG**	**GC**	**CC**					
**rs884861**	**Cases**	0.161	0.435	0.404	248	0.16	0.38	0.05	
	**Controls**	0.220	0.440	0.340	273		0.44		G allele, 0.43

^a^MAF = Minor allele frequency.

^b^Heterozygous GA genotype versus homozygous AA genotype only as no homozygous GG individuals were detected.

## Discussion

Susceptibility to migraine is conferred by exposure to intrinsic and environmental triggering factors and genetics. Pinpointing SNPs associated with disease is one of the goals of GWAS and one of many approaches used to dissect the genetic basis of migraine. This study was undertaken to follow up findings from a pGWAS we previously conducted in the Norfolk Island pedigree which implicated four SNPs in the RNA editing gene *ADARB2* in migraine, based on statistical significance [11]. These four SNPs, and others, in the RNA editing genes *ADARB1* and *ADARB2*, were further investigated in an Australian migraine case–control population because they fit criteria for migraine neuropathology, i.e. a) are expressed in the brain or CNS, b) regulate neurological pathways (e.g. neurotransmitters) and c) are plausibly related to migraine neuropathology (e.g. cellular hyperexcitability, ion channel disruption). Furthermore, retyping top-ranking SNPs from GWAS data in independent case-controls cohorts is important to determine the validity of GWAS findings and ascertain risk within different population groups.

To date only a few association studies have investigated SNPs in the RNA editing genes *ADARB1* and *ADARB*2 and none have been studied in migraine previously. Interestingly, a GWAS of US centenarians supports a role for RNA editors as important regulators of aging in humans. SNPs in *ADARB2* were found to be associated with longevity and these findings were replicated in three independent cohorts of different genetic backgrounds [27]. Amore et al. [28] identified a common neutral SNP in three out of seven patients with bipolar disorder in the *ADARB1* gene, but no other major alterations. In a separate study, Kostyrko et al. [29] analysed the coding sequence of *ADARB1* and its association with bipolar affective disorder and did not find any mutations except one already known transition. Oguro et al. [30] identified a longevity-associated SNP in *ADARB2* rs2805533, which may modulate human longevity by regulating metabolic factors such as abdominal obesity and lipid profiles.

RNA editing is a physiologically important and conserved process necessary for proper development and functioning of neuronal cells. The importance of this process is demonstrated by mice deficient in the RNA-editing enzyme *ADARB1* which die approximately 20 days post-birth and show early onset epilepsy [31]. This phenotype results from under editing of a critical position that determines calcium permeability in glutamate receptors of excitatory neurons [31]. *ADARB1* plays an important role in ensuring neurotransmitter receptor transcripts are properly edited at respective points [14]. The particular editing function of *ADARB1* upon the AMPA glutamate receptor subunit (*GRIA2*) pre-mRNA makes the investigation of *ADARB1* in migraine interesting, because glutamate is a major mediator in the CNS and its regulation has been studied as a possible mechanism causing migraine.

Although *ADARB2* is catalytically inactive it has been shown *in vitro* that it can act as a competitor of *ADAR* (also known as *ADAR1*) and *ADARB1* by binding to the same transcripts and thus *ADARB2* can decrease the efficiency of other RNA editing enzymes by preventing them from deaminating their substrate RNAs in regions of the brain where they are co-expressed [32]. *ADARB2* may therefore be reducing the total number of edited transcripts via a competitive mechanism, which in turn may lead to poor ionic conductance and synaptic dysfunction in the CNS. One example that supports this hypothesis is perturbed A-to-I editing of the mRNA encoding the *GRIA2* subunit of glutamate AMPA receptors which manifests in the disease amyotrophic lateral sclerosis (ALS) [33]. Functional studies are needed to further investigate the activity of *ADARB2 in vivo*.

In this study we tested four SNPs in the *ADARB1* gene and eight SNPs in the *ADARB2* for association with migraine susceptibility. Results of *χ*^2^ analysis indicated that neither genotype nor allele frequency distributions for the migraine and control groups were significantly different after Bonferroni correction for multiple testing. Although we were unable to replicate our findings from the Norfolk Island population implicating four *ADARB2* SNPs in migraine in an Australian Caucasian population, it does not exclude a role for *ADARB2* in migraine in the unique Norfolk Island population isolate. This may be because rare variants that are over-represented in the unique pedigree structure of the Norfolk Island population contributed to the initial finding and that the common variants selected for this study are not relevant in the case–control population analysed here. Fine-mapping and sequencing for rare variants in the Norfolk Island samples could shed further light on this.

## Conclusion

In this study we found no evidence for SNPs in the RNA editing genes *ADARB1* and *ADARB2* to be associated with increased risk of migraine in an Australian Caucasian population. This result is in contrast to a pGWAS study of Norfolk Island which implicated a 22 kb haploblock region in *ADARB2* in migraine. Genetically isolated populations may carry unique genetic characteristics that are not always replicated in case–control cohorts. Nevertheless, both types of populations are useful to study the genetic structure underlying disease, providing direction to the investigation of susceptibility genes and genetic pathways. Considering that common migraine is caused by interactions of multiple loci, identifying and validating genetic factors that influence the disorder remains an important undertaking that may lead to improved strategies for management and treatment.

## Abbreviations

*ADARB1*: Adenosine deaminase, RNA-specific, B1
*ADARB2*: Adenosine deaminase, RNA specific, B2
ALS: Amyotrophic lateral sclerosis
A-to-I: Adenosine-to-Inosine
CEU: Utah residents with Northern and Western European ancestry
CNS: Central Nervous System
CSD: Cortical Spreading Depression
*DBH*: Dopamine Beta Hydroxylase
DSH: Dyschromatosis symmetrica hereditaria
FHM: Familial Hemiplegic Migraine
*GRIA2*: Glutamate receptor, ionotropic, AMPA2
*GRM7*: Glutamate receptor, metabotropic 7
*HTR7*: 5-hydroxytryptamine serotonin receptor 7 adenylate cyclase-coupled
HWE: Hardy-Weinberg Equilibrium
IHS: International Headache Society
MA: Migraine with Aura
MO: Migraine without Aura
LD: Linkage disequilibrium
pGWAS: Pedigree-based genome wide association study
*SLC6A3*: Solute carrier family 6 (neurotransmitter transporter), member 3
SNPs: Single nucleotide polymorphisms
